# Evaluation of the ABCG2 Charge Model in Protein–Ligand Binding Free-Energy Calculations

**DOI:** 10.1021/acs.jcim.5c02161

**Published:** 2025-10-30

**Authors:** Sudarshan Behera, Vytautas Gapsys, Bert L. de Groot

**Affiliations:** † Computational Biomolecular Dynamics Group, 28282 Max Planck Institute for Multidisciplinary Sciences, Göttingen 37077, Germany; ‡ In Silico Discovery Janssen Research & Development, 50148 Janssen Pharmaceutica N.V., Turnhoutseweg 30, 2340 Beerse, Belgium

## Abstract

Accurate binding free energy prediction is vital for drug design, motivating the assessment of new force field models. Here, we evaluate the ABCG2 charge model with nonequilibrium alchemical free-energy simulations. GAFF2/ABCG2 achieves higher hydration free energy accuracy but does not outperform GAFF2/AM1-BCC for protein–ligand binding free energy. Both charge models exhibit comparable accuracy and compound ranking across targets, indicating that property-specific force field optimization does not guarantee improved related-property performance.

## Graphical abstract

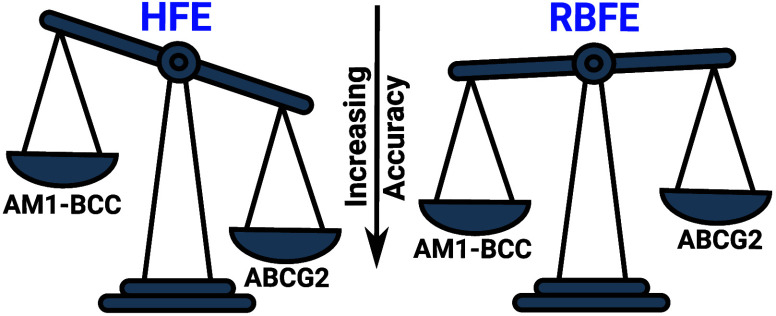

## 1 Introduction

Predicting small-molecule binding free energies to biomolecular targets is central to drug design.^1–3^ Molecular dynamics, in particular, alchemical free-energy calculations^4,5^ allows rigorous estimation of these affinities, using protocols such as free-energy perturbation^6^ or thermodynamic integration.^7^ These methods are widely used in both benchmarking and drug discovery.^8–11^

Accurately predicting binding free energies depends on the reliable parametrization of small molecule interactions, which is determined by the choice of force field. Several force fields are commonly used, including GAFF2,^12^ CGenFF,^13^ OpenFF,^14^ and OPLS-3/4,^15,16^ all of which have demonstrated good performance on benchmark datasets. However, outliers in these studies are often linked to suboptimal force field parametrization.^10,17,18^ A critical component influencing prediction accuracy is the assignment of partial atomic charges. To address limitations in practical charge assignments, strategies such as employing polarizable force fields have been explored,^19,20^ although empirical evidence suggests that the latter improves accuracy only in specific cases.^19,20^

The combination of GAFF2 parameters with AM1-BCC partial charges is shown to yield robust performance in large-scale hydration free-energy (HFE) calculations^21,23^ and in both relative and absolute protein–ligand binding free-energy studies.^10,24^ For instance, on the widely used FreeSolv dataset,^25^ the GAFF2/AM1-BCC approach achieves a root-mean-square error (RMSE) of 1.71 kcal/mol.^21^ Recently, He et al. introduced a novel bond charge correction scheme, ABCG2 (AM1-BCC-GAFF2),^21,23^ which substantially improves predictive accuracy for HFEs, lowering the RMSE to 0.99 kcal/mol. The ABCG2 charge model is optimized by generating new BCC terms for certain functional groups to achieve highly accurate HFE.^23^ Beyond hydration, the GAFF2/ABCG2 combination has also shown excellent transferability for other properties, including transfer free energy of solutes from water to organic solvents, as well as density and heat of vaporization of neat organic liquids.^21^ A recent study verified the accuracy of ABCG2 for HFE calculations across 11 small molecules, leading to speculation about the advantage of using this charge model in protein–ligand binding free-energy calculations.^26^ Nevertheless, the performance of this new charge model in the complex environment of proteins remains to be evaluated.

The goal of this report is to assess the performance of the GAFF2/ABCG2 parametrization in the protein–ligand relative binding free-energy (RBFE) simulations using nonequilibrium alchemy^27–29^ (refer to section S1 for details regarding the methodology). Our findings confirm that GAFF2/ABCG2 improves HFE prediction, achieving an RMSE of approximately 1 kcal/mol—a significant improvement over the GAFF2/AM1-BCC combination. However, when the method was applied to RBFE calculations, no improvement was observed. Both GAFF2/AM1-BCC and GAFF2/ABCG2 (in combination with two protein force fields, AMBER99SB*-ILDN^30,31^ and AMBER14SB^32^) show comparable accuracy in RBFE calculations and in ranking drug candidates by free energy.

## 2 Results and Discussion

We first validated our protocol on hydration free-energy (HFE) calculations using the FreeSolv database,^25^ which comprises 642 organic molecules. Our results are in close agreement with those previously reported by He et al. Statistical measures such as AUE, RMSE, Pearson’s *r*, Spearman’s ρ, and Kendall’s τ show strong concordance between the current study (pmx) and the results of He et al.^21^ for both GAFF2/AM1-BCC and GAFF2/ABCG2 parameter sets (see Figures 1a and 1b, as well as Figures S1 and S2). For example, the RMSE, reported by He et al.,^21^ between the estimated and experimental HFE with GAFF2/ABCG2 is 0.98 [0.81, 1.20] kcal/mol, while our corresponding estimate is 1.00 [0.86, 1.17] kcal/mol. Square brackets are used to show 95% confidence throughout the manuscript, and GAFF2 parameters are used for ligands unless otherwise stated.

**Figure 1:**
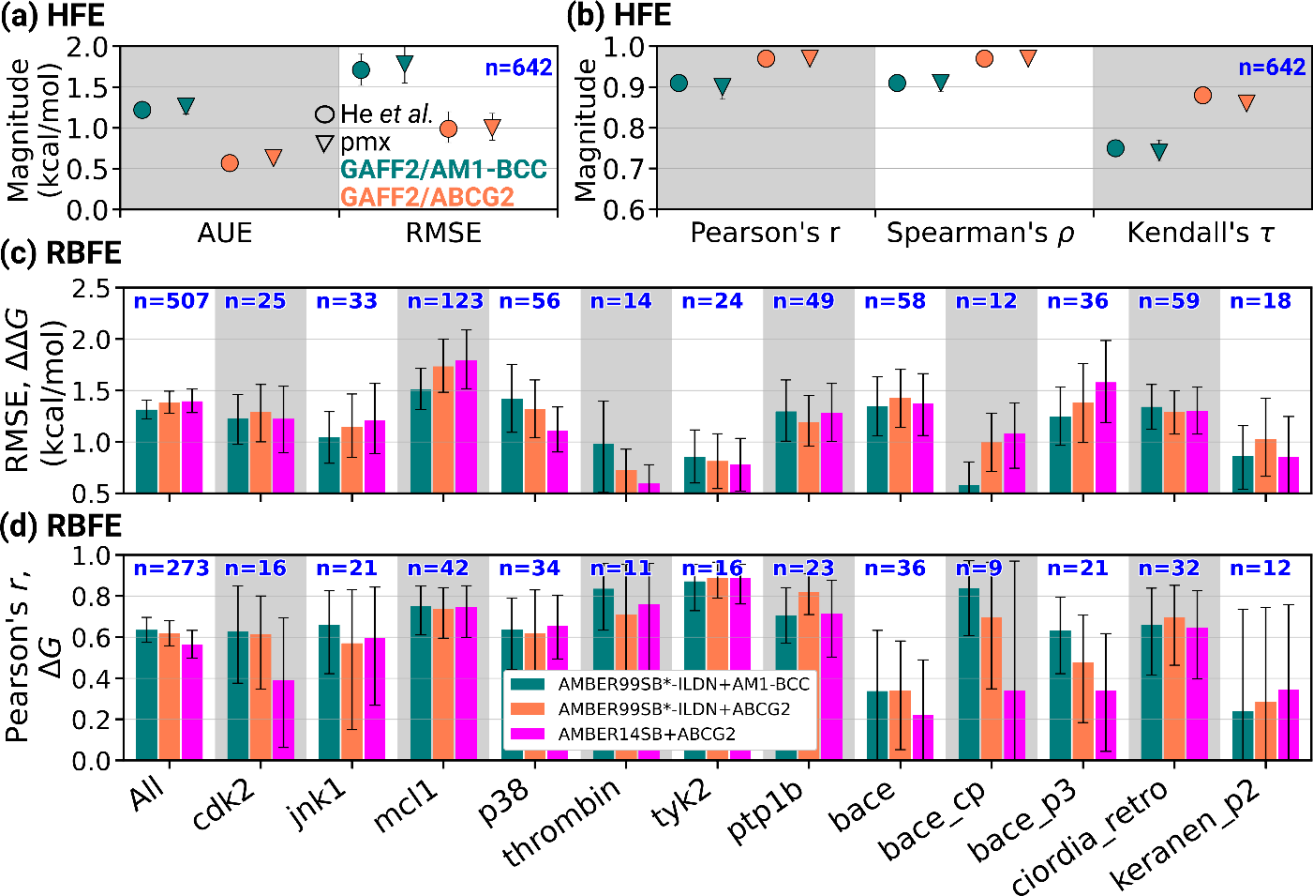
(a, b) Comparison of accuracy and correlation metrics between previous results from He et al.^21^ and those from the current study (pmx) for HFE calculations, including statistical measures such as AUE (absolute-unsigned-error), RMSE, Pearson’s *r*, Spearman’s ρ, and Kendall’s τ. Panel b shares the same legends mentioned in panel a. (c) RMSE of protein–ligand RBFE predictions (ΔΔ*G*) relative to experimental data, assessed for different force field combinations. (d) Comparisons of the Pearson’s correlation coefficients (*r*) between calculated and experimental Δ*G* values. Δ*G* values were obtained from RBFE estimates via maximum likelihood estimation.^22^ Panels (c) and (d) share the same legend and *x*-axis labels as in panel (d). “bace_cp” and “bace_p3” are abbreviations for ‘bace_ciordia_prospective” and “bace_p3_arg368_in”, respectively. The correlation coefficient for “All” is computed as a weighted average of the values obtained for each target, with the weight being the number of data points (“*n*”). The error bars are the 95% confidence interval. Spearman’s ρ and Kendall’s τ for RBFE are presented in Figure S15. The RMSE on ΔΔ*G* and Δ*G* and Pearson’s *r* values are also tabulated in Tables S1, S2, and S3. *p*-values obtained from a paired Student’s *t*-test for ΔΔ*G* estimates obtained using different force field comparisons are tabulated in Table S4.

Following our initial validation, we conducted RBFE calculations using a subset of the dataset prepared by the OpenFE consortium,^33^ consisting of 12 protein targets, 273 ligands, and 507 ligand perturbations (from the work of Ross et al.^11^). Our results (ΔΔ*G*) indicate that the ABCG2 charge model does not provide an improvement in relative binding free-energy predictions over the AM1-BCC model (see Figure 1c and Figure S3). Across the full dataset, the RMSE values are 1.31 [1.22, 1.41], 1.38 [1.28, 1.49], and 1.39 [1.28, 1.51] kcal/mol for AMBER99SB*-ILDN+GAFF2/AM1-BCC, AMBER99SB*-ILDN+GAFF2/ABCG2, and AMBER14SB+GAFF2/ABCG2, respectively (Table S1). The dataset splitted as ‘jacs_set’^34^ and “janssen_bace” ^35,36^ is also presented in Figures S4 and S5, respectively. For all of the individual targets, all three force field combinations yield similar RMSE with statistically nonsignificant differences. There are some targets, such as thrombin and bace_cp, for which the RMSE differences are as large as 0.3 kcal/mol, but statistically not significant (see Figure 1c, as well as Figures S6 and S7, and Table S1). A paired Student’s *t*-test between the ΔΔ*G* estimates with AMBER99SB*-ILDN+GAFF2/AM1-BCC and AMBER99SB*-ILDN+GAFF2/ABCG2 results in a *p* value of 0.21 (Table S4). Additionally, the distributions of standard errors for the estimated HFEs and RBFEs are similar for both charge models (Figures S8 and S9).

One of the main goals in drug discovery applications is to reliably rank different candidate compounds. To this end, the calculated ΔΔ*G* values were converted to absolute binding free energies (Δ*G*), using the maximum likelihood estimation method implemented in Cinnabar,^22^ and compared against experiments in Figures S10–S14. We assessed the ranking performance of ligands across different targets, as shown in Figure 1c and Figure S15. Consistent with the trends observed for the accuracy of ΔΔ*G*, the accuracy on Δ*G* and Pearson’s *r* (also Spearman’s ρ and Kendall’s τ) for ligand rankings are similar across all targets for both GAFF2/ABCG2 and GAFF2/AM1-BCC parametrizations. The RMSEs on the Δ*G* across the full dataset are 0.97 [0.88, 1.07], 1.05 [0.94, 1.16], and 1.15 [1.01, 1.29] kcal/mol, respectively, using AMBER99SB*-ILDN+GAFF2/AM1-BCC, AMBER99SB*-ILDN+GAFF2/ABCG2, and AMBER14SB+ GAFF2/ABCG2 (Figure S10, Table S2). For the “jacs_set”,^34^ the RMSEs on the Δ*G* using AMBER99SB*-ILDN+GAFF2/AM1-BCC, AMBER99SB*-ILDN+GAFF2/ABCG2, and AMBER14SB+GAFF2/ABCG2 are 0.97 [0.86, 1.09], 0.97 [0.85, 1.12], and 1.00 [0.87, 1.13] kcal/mol, respectively, (Figure S11). Similarly, for the “janssen_bace”,^35,36^ the same sequence of force field combinations yields RMSEs of 0.97 [0.82, 1.12], 1.21 [1.00, 1.43], and 1.47 [1.15, 1.78] kcal/mol, respectively (Figure S12). For comparison, FEP+ leads to RMSEs of 0.76 [0.69, 0.83] kcal/mol for the full dataset, 0.73 [0.65, 0.80] kcal/mol for the “jacs_set”, and 0.83 [0.70, 0.98] kcal/mol for “janssen_bace”.^11^

We further analyzed the RMSEs on ΔΔ*G* obtained with both charge models for various functional group perturbations (see Figure 2, as well as Figure S16). Our observations show that the difference in RMSEs for all the functional groups, with AM1-BCC and ABCG2 charges, is statistically nonsignificant. Table S5 lists *p*-values for comparison of different functional group perturbations with all three force field combinations. Although it is nonsignificant, GAFF2/ABCG2 agrees with experiments better than GAFF2/AM1-BCC in some cases, as highlighted in Figure 2b, whereas the agreement is worse in some other cases (Figure 2c).

**Figure 2:**
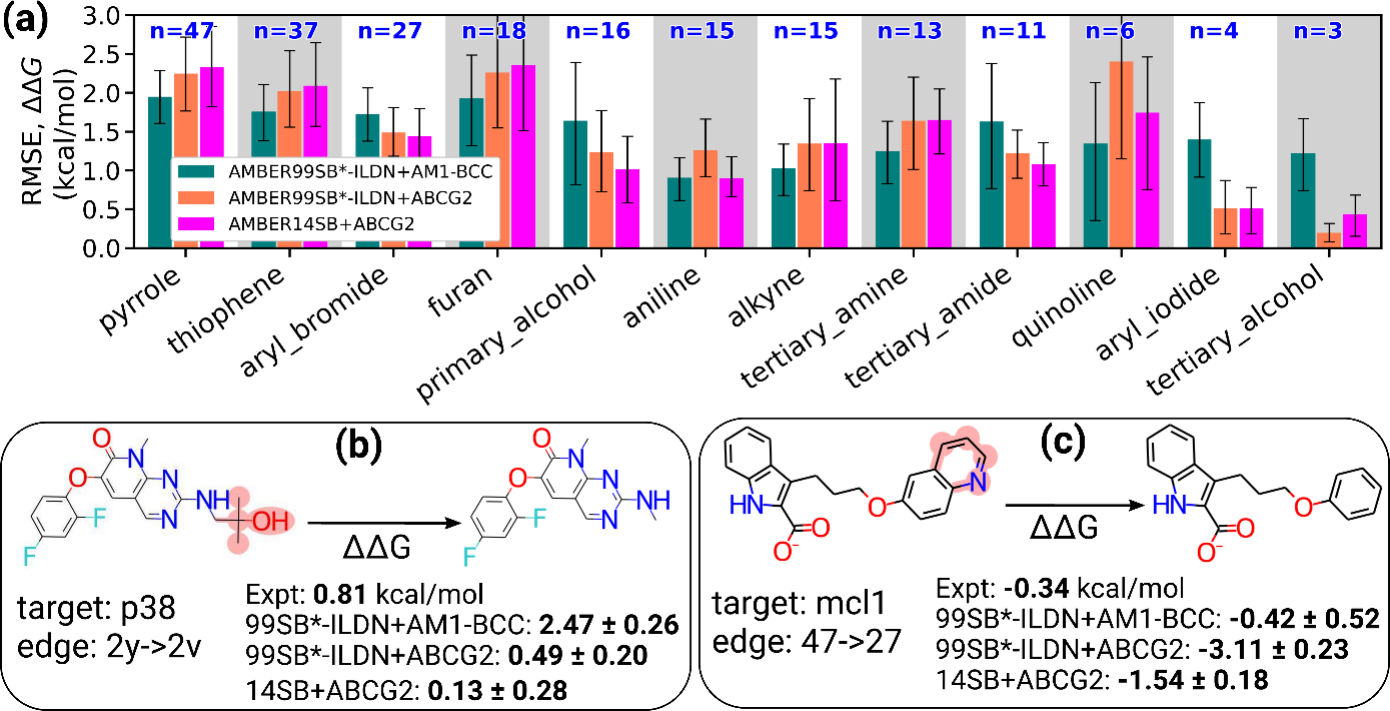
(a) RMSEs for subsets of ligand transformations involving various functional groups, highlighting only those groups where the RMSE difference between AMBER99SB*-ILDN+AM1-BCC and AMBER99SB*-ILDN+ABCG2 is >1 kJ/mol (0.24 kcal/mol). Additional functional group analyses are listed in Figure S16. Note that the same transformation may contribute to multiple categories (e.g., a biphenyl group perturbation is counted in both biphenyl and aromatic classes). (b) Shown is a case with a tertiary alcohol, where ABCG2 significantly improved RBFE prediction, matching the experimental value closely. (c) In contrast, a quinoline example illustrates that switching to ABCG2 substantially worsened RBFE prediction accuracy relative to AM1-BCC. The reported uncertainties in panels (b) and (c) are standard errors calculated as described in section S1.3.

The limited transferability of the ABCG2 charge model to protein–ligand binding free-energy calculations may be due to its BCC parameters being specifically optimized for hydration free energy (HFE) accuracy, making them insufficiently general for the complex and heterogeneous environments of protein binding pockets. While GAFF2/ABCG2 provides accurate predictions for hydration and solvent transfer free energies, it does not improve the protein–ligand binding free energy in combination with two current protein force fields. This suggests that improving binding free-energy predictions may require further optimizing ligand parameters for protein–ligand interactions or developing a protein force field tailored for compatibility with GAFF2/ABCG2 ligand parameters. Given the conformation dependence of AM1-BCC and ABCG2 charges,^21,37^ and the sensitivity of free-energy calculations to binding poses^18^ and sampling protocols,^38^ our findings may vary for different targets or ligands under alternative methodologies. Nonetheless, based on our large-scale results, the GAFF2/ABCG2 model does not provide a statistically significant improvement in accuracy, compared to GAFF2/AM1-BCC, and this conclusion is likely robust across broader applications.

## 3 Conclusions

The results of this study highlight a fundamental challenge in computational chemistry: optimizing a force field or charge model for one molecular property does not guarantee improved performance for other, even closely related, properties. While our validation confirms that the GAFF2/ABCG2 combination significantly enhances the accuracy of HFE predictions—reaching RMSEs as low as 1 kcal/mol, this improvement does not translate to protein–ligand relative binding free-energy calculations. Our extensive RBFE benchmarking reveals that ABCG2 offers no overall advantage over the well-established AM1-BCC charges. Both models yield comparable accuracy across a wide array of ligand transformations and functional groups with only minor, statistically nonsignificant differences. Overall, our results indicate that new parameter sets should be validated across diverse systems before being regarded as broadly useful for complex applications, such as drug discovery.

## Supplementary Material



## Data Availability

The input files, ΔΔ*G*, and Δ*G* values can be found at https://github.com/deGrootLab/abcg2_evaluation/. GROMACS and pmx are available freely at https://www.gromacs.org/ and https://github.com/deGrootLab/pmx, respectively.

## Notes

Methodology, scatter plots of experiments vs simulations, and additional figures/tables/data (PDF)

## References

[1] Beveridge D. L., Dicapua F. M. (1989). Free energy via molecular simulation: applications
to chemical and biomolecular systems. Annu.
Rev. Biophys. Biophys. Chem..

[2] Zhou H.-X., Gilson M. K. (2009). Theory of free energy
and entropy in noncovalent binding. Chem. Rev..

[3] Cournia Z., Allen B., Sherman W. (2017). Relative binding
free energy calculations
in drug discovery: recent advances and practical considerations. J. Chem. Inf. Model..

[4] Chipot, C. ; Pohorille, A. Free Energy Calculations: Theory and Applications in Chemistry and Biology, Vol. 86; Springer Science & Business Media, 2007.

[5] Chodera J.
D., Mobley D. L., Shirts M. R., Dixon R. W., Branson K., Pande V. S. (2011). Alchemical
free energy methods for drug discovery:
progress and challenges. Curr. Opin. Struct.
Biol..

[6] Zwanzig R. W. (1954). High-temperature
equation of state by a perturbation method. I. Nonpolar gases. J. Chem. Phys..

[7] Kirkwood J. G. (1935). Statistical
mechanics of fluid mixtures. J. Chem. Phys..

[8] Schindler C. E., Baumann H., Blum A., Bose D., Buchstaller H.-P., Burgdorf L., Cappel D., Chekler E., Czodrowski P., Dorsch D. (2020). Large-scale
assessment of binding free energy calculations
in active drug discovery projects. J. Chem.
Inf. Model..

[9] Gapsys V., Pérez-Benito L., Aldeghi M., Seeliger D., Van Vlijmen H., Tresadern G., De Groot B. L. (2020). Large scale relative protein ligand
binding affinities using non-equilibrium alchemy. Chem. Sci..

[10] Hahn D. F., Gapsys V., de Groot B. L., Mobley D. L., Tresadern G. (2024). Current state
of open source force fields in protein-ligand binding affinity predictions. J. Chem. Inf. Model..

[11] Ross G. A., Lu C., Scarabelli G., Albanese S. K., Houang E., Abel R., Harder E. D., Wang L. (2023). The maximal and current accuracy
of rigorous protein-ligand binding free energy calculations. Commun. Chem..

[12] Wang J., Wolf R. M., Caldwell J. W., Kollman P. A., Case D. A. (2004). Development
and testing of a general amber force field. J. Comput. Chem..

[13] Vanommeslaeghe K., Hatcher E., Acharya C., Kundu S., Zhong S., Shim J., Darian E., Guvench O., Lopes P., Vorobyov I., Mackerell A. D. (2010). CHARMM general
force field: A force field for drug-like molecules compatible with
the CHARMM all-atom additive biological force fields. J. Comput. Chem..

[14] Boothroyd S., Behara P. K., Madin O. C., Hahn D. F., Jang H., Gapsys V., Wagner J. R., Horton J. T., Dotson D. L., Thompson M. W. (2023). Development and benchmarking of open force
field 2.0: the Sage small molecule force field. J. Chem. Theory Comput..

[15] Harder E. (2016). OPLS3: a force field providing broad coverage of drug-like
small
molecules and proteins. J. Chem. Theory Comput..

[16] Lu C., Wu C., Ghoreishi D., Chen W., Wang L., Damm W., Ross G. A., Dahlgren M. K., Russell E., Von Bargen C. D. (2021). OPLS4: Improving force field accuracy on challenging regimes of chemical
space. J. Chem. Theory Comput..

[17] Aldeghi M., Heifetz A., Bodkin M. J., Knapp S., Biggin P. C. (2017). Predictions
of ligand selectivity from absolute binding free energy calculations. J. Am. Chem. Soc..

[18] Behera S., Hahn D. F., Wilson C. J., Marsili S., Tresadern G., Gapsys V., de Groot B. L. (2025). Quantification of the Impact of Structure
Quality on Predicted Binding Free Energy Accuracy. J. Chem. Inf. Model..

[19] Nawrocki G., Leontyev I., Sakipov S., Darkhovskiy M., Kurnikov I., Pereyaslavets L., Kamath G., Voronina E., Butin O., Illarionov A. (2022). Protein-Ligand Binding
Free-Energy Calculations with ARROW- A Purely First-Principles Parameterized
Polarizable Force Field. J. Chem. Theory Comput..

[20] Damm, W. OPLS5: Addition of Polarizability and Improved Treatment of Metals. 2024,.

[21] He X., Man V. H., Yang W., Lee T.-S., Wang J. (2025). ABCG2: AMilestone
Charge Model for Accurate Solvation Free Energy Calculation. J. Chem. Theory Comput..

[22] Gowers, R. ; Macdonald, H. B. ; Henry, M. ; Travitz, A. ; Alibay, I. ; dfhahn; Pulido, I. ; Mitchell, J. A. ; Zhang, I. ; Chodera, J. ; Dotson, D. L. ; glass w; Baumann, H. anna OpenFreeEnergy/cinnabar: v0.5.0. 2025; 10.5281/zenodo.15678719.

[23] He, X. ; Man, V. H. ; Yang, W. ; Lee, T.-S. ; Wang, J. A fast and high-quality charge model for the next generation general AMBER force field. J. Chem. Phys. 2020, 153. 10.1063/5.0019056 PMC7728379 32962378

[24] Khalak Y., Tresadern G., Aldeghi M., Baumann H. M., Mobley D. L., de Groot B. L., Gapsys V. (2021). Alchemical absolute protein–ligand
binding free energies for drug design. Chem.
Sci..

[25] Mobley D.
L., Guthrie J. P. (2014). FreeSolv:
a database of experimental and calculated
hydration free energies, with input files. J.
Comput.-Aided Mol. Des..

[26] Orlandi M., Geng Y., Macchiagodena M., Pagliai M., Procacci P. (2025). Solvation
Free Energies of Drug-like Molecules via Fast Growth in an Explicit
Solvent: Assessment of the AM1-BCC, RESP/HF/6–31G*, RESP-QM/MM,
and ABCG2 Fixed-Charge Approaches. J. Chem.
Theory Comput..

[27] Jarzynski C. (1997). Nonequilibrium
equality for free energy differences. Phys.
Rev. Lett..

[28] Crooks G. E. (1999). Entropy
production fluctuation theorem and the nonequilibrium work relation
for free energy differences. Phys. Rev. E.

[29] Behera, S. ; Wilson, C. J. ; Schmidt, L. ; de Groot, B. L. Free Energy Simulations to Quantitatively Study Biomolecule Stability and Binding. ChemRxiv Preprints 2025, 10.26434/chemrxiv-2025-2zbd8.

[30] Lindorff-Larsen K., Piana S., Palmo K., Maragakis P., Klepeis J. L., Dror R. O., Shaw D. E. (2010). Improved
side-chain
torsion potentials for the Amber ff99SB protein force field. Proteins: Struct., Funct., Bioinf..

[31] Best R. B., Hummer G. (2009). Optimized molecular
dynamics force fields applied to
the helix-coil transition of polypeptides. J.
Phys. Chem. B.

[32] Maier J. A., Martinez C., Kasavajhala K., Wickstrom L., Hauser K. E., Simmerling C. (2015). ff14SB: improving the accuracy of
protein side chain and backbone parameters from ff99SB. J. Chem. Theory Comput..

[33] Gowers, R. J. ; Alibay, I. ; Swenson, D. W. ; Henry, M. M. ; Ries, B. ; Baumann, H. M. ; Eastwood, J. R. B. The Open Free Energy library. 2023; 10.5281/zenodo.8344248.

[34] Wang L. (2015). Accurate and reliable
prediction of relative ligand binding potency
in prospective drug discovery by way of a modern free-energy calculation
protocol and force field. J. Am. Chem. Soc..

[35] Ciordia M., Pérez-Benito L., Delgado F., Trabanco A. A., Tresadern G. (2016). Application
of free energy perturbation for the design of BACE1 inhibitors. J. Chem. Inf. Model..

[36] Keranen H., Pérez-Benito L., Ciordia M., Delgado F., Steinbrecher T. B., Oehlrich D., Van Vlijmen H. W., Trabanco A. A., Tresadern G. (2017). Acylguanidine
beta secretase 1 inhibitors: a combined experimental and free energy
perturbation study. J. Chem. Theory Comput..

[37] Osato M., Baumann H. M., Huang J., Alibay I., Mobley D. L. (2025). Evaluating
the Functional Importance of Conformer-Dependent Atomic Partial Charge
Assignment. J. Comput. Chem..

[38] Rizzi A., Jensen T., Slochower D. R., Aldeghi M., Gapsys V., Ntekoumes D., Bosisio S., Papadourakis M., Henriksen N. M., De Groot B. L. (2020). The SAMPL6 SAMPLing
challenge: assessing the reliability and efficiency of binding free
energy calculations. J. Comput.-Aided Mol. Des..

