# Supplementary material for: Evaluation of the ABCG2 Charge Model in Protein–Ligand Binding Free-Energy Calculations
Source: J Chem Inf Model. 2025 Oct 30;65(21):11505–9. doi: 10.1021/acs.jcim.5c02161 (PMC12606647; doi:10.1021/acs.jcim.5c02161)
Supplement: Supplementary file 1 [file ci5c02161_si_001.pdf]

Supporting Information
Evaluation of the ABCG2 charge model in protein–ligand binding free energy calculations

Sudarshan Behera,[†] Vytautas Gapsys,[*,‡] and Bert L. de Groot[*,†]

†*Computational Biomolecular Dynamics Group, Max Planck Institute for Multidisciplinary Sciences, Göttingen 37077, Germany*

‡*In Silico Discovery, Janssen Research & Development, Janssen Pharmaceutica N. V., Turnhoutseweg 30, 2340 Beerse, Belgium*

E-mail: vgapsys@its.jnj.com; bgroot@gwdg.de

S1 Methodology

S1.1 Solvation free energy

The SMILES strings of the 642 solutes were obtained from the FreeSolv database,[1] and 3D structures were constructed using rdkit.[2] GAFF2 parameters, along with AM1-BCC and ABCG2 partial charges, were assigned using Antechamber[3] and ACPYPE,[4] following the protocol detailed by He *et al.*[5] The steps for extracting GAFF2/AM1-BCC parameters are as follows,

```
antechamber -i ${lig}.sdf -fi sdf -o ${lig}.mol2 -fo mol2 -c bcc -at gaff2
-nc ${net_charge} -j 5
parmchk2 -i ${lig}.mol2 -f mol2 -o ${lig}.frcmod -s gaff2
acpype -i ${lig}.mol2 -n ${net_charge} -a gaff2 -c user
```

The ${lig} and ${net_charge} refer to the solute name and the total charge of the solute. The same protocol with "antechamber -c abcg2" was followed to get GAFF2/ABCG2 parameters.

Hydration free energy (HFE) calculations were performed within a nonequilibrium alchemical framework. Each molecule was centred within a dodecahedral simulation box, ensuring that the edges were at least 15 Å away from any atom of the solute. The systems were subsequently solvated with the TIP3P water model. Na^+ and Cl^- ions (with Joung & Cheatham's ion parameters[6]) were added to attain neutrality and reach a final concentration of 0.15 M. Following solvation and ion addition, each system underwent energy minimisation, 10 ps of equilibration in the NVT ensemble, and a 6 ns production run in the NPT ensemble. These simulation protocols were carried out for both the coupled (fully interacting) and decoupled (noninteracting) states of the solute, controlled by the alchemical variable λ. The initial 2.25 ns of the production trajectory were discarded as equilibration, and 80 configurations were sampled from the remaining 3.75 ns. Nonequilibrium transitions were then initiated from these frames, with λ varied from 0 to 1 and *vice versa.* Each nonequilibrium switching trajectory was carried out for 50 ps. The entire procedure from energy

minimization was carried out three times, and the ΔG reported is the average of the three estimates. In total, 60 ns (20 ns per replica) was spent on an HFE calculation.

S1.2 Protein ligand relative binding free energy (RBFE)

For the RBFE calculations, initial structures for the "jacs_set"[7] and "janssen_bace"[8,9] datasets, as prepared by OpenFE,[10,11] were employed in this study. Combined, these datasets comprise 12 protein targets and 273 ligands. The ligand transformation map (edges) is taken from Ross *et al.*,[12] considering only one conformation and protonation state of ligands, leading to a total of 507 ligand perturbations. All ligands underwent the same parameterization protocol described for the solvation free energy calculations to generate GAFF2 parameters with both AM1-BCC and ABCG2 partial charges. Atom mapping and the generation of hybrid structures and topologies for each ligand pair were performed using pmx.[13] The resulting hybrid structures and topologies were then integrated into their corresponding protein targets, with the coupling variable λ controlling the alchemical transformation between ligand A ($\lambda = 0$) and ligand B ($\lambda = 1$). The proteins are modelled using the AMBER99SB*-ILDN[14–16] and AMBER14SB[17] force fields. The free energy change of ligand transformation was estimated in both the protein and water branch using the same series of equilibrium and nonequilibrium molecular dynamics simulations as outlined for the solvation free energy calculations, following the protocols described in previous reports.[18,19] The reported $\Delta\Delta G$ is thus the difference between the estimated ΔGs from protein and water branches, and averaged over three repeats.

S1.3 Analysis

The analysis of free energy calculations was carried out using pmx. Work values for the 50 ps alchemical transitions were computed by integrating the change in Hamiltonian with respect to the coupling parameter ($dH/d\lambda$) along the non-equilibrium trajectories. Work distributions from the forward (λ: 0 to 1) and reverse (λ: 1 to 0) transitions were then used

to estimate free energy differences via the maximum likelihood estimator,[20] based on the Crooks Fluctuation Theorem.[21] The reported $\Delta\Delta G$ values represent the average from three independent replicas. Uncertainties for statistical metrics, such as average unsigned error (AUE), root-mean-squared error (RMSE), Pearson's r, Kendall's τ, and Spearman's ρ are given as 95% confidence intervals, determined using bootstrapping unless noted otherwise.

The uncertainties associated with ΔG and $\Delta\Delta G$ calculations follow procedures described previously,[19] (i) for every leg (protein and water) and every replica, the 80 forward and 80 reverse work values were bootstrapped n_{boot} times (bootstrapped samples) to calculate ΔG_b (bootstrapped ΔG values). (ii) a Normal distribution was constructed using the previously calculated ΔG value (using the 80 forward and 80 reverse work values without bootstrapping) and standard deviation of the ΔG_b values, from which n_{boot} samples were extracted.

$$\Delta G_{bi} \sim \mathcal{N}(\Delta G, \sigma_{\Delta G_b}), \quad i = 1, \ldots, n_{boot} \tag{1}$$

The n_{boot} ΔG_{bi} values from all the replica for a leg (protein/water) are pooled together into a set (S_l), for which the standard error (SE) on ΔG for that specific leg is estimated as

$$\mathrm{SE}(l) = \sqrt{\frac{\mathrm{variance}(\mathcal{S}_l)}{n_{\mathrm{replica}}}}, \quad l = \mathrm{protein/water} \tag{2}$$

$$\mathcal{S}_l = \left\{ \left\{ \Delta G_{b,l,(k)}^{(i)} \right\}_{i=1}^{n_{boot}} \right\}_{k=1}^{n_{\mathrm{replica}}}, \quad b : \textit{bootstrapped} \tag{3}$$

The final uncertainty on $\Delta\Delta G$ is calculated as,

$$SE(\Delta\Delta G) = \sqrt{\mathrm{SE}(protein)^2 + \mathrm{SE}(water)^2} \tag{4}$$

The $n_{replica}$ and n_{boot} are 3 and 1000, respectively.

S1.4 Additional details

Energy minimization was performed using the steepest descent algorithm,[22] with a maximum force tolerance set to 100 kJ mol^{-1} nm^{-1}. Molecular dynamics simulations utilized a stochastic dynamics thermostat and a Parrinello-Rahman barostat,[23] with coupling time constants of 2 ps and 5 ps, respectively, in combination with the leap-frog stochastic dynamics integrator.[24] A timestep of 4 fs was employed, facilitated by using a hydrogen mass repartitioning factor of 3. All bonds were constrained using the LINCS algorithm,[25] while long-range electrostatics were treated with the Particle Mesh Ewald (PME) method[26] and a real-space cutoff of 11 Å. van der Waals interactions were smoothly switched between 10 and 11 Å and dispersion corrections to both energy and pressure were applied. During the alchemical transitions, non-bonded interactions were managed using the 'gapsys' soft-core potential[27] implemented in GROMACS, with default parameters. The value of $dH/d\lambda$ was recorded at every integration step throughout the transitions. All simulations were performed using GROMACS-2022.6.[28] GAFF2 parameters with AM1-BCC or ABCG2 charges are used for ligands, unless stated otherwise.

S2 Supplementary figures

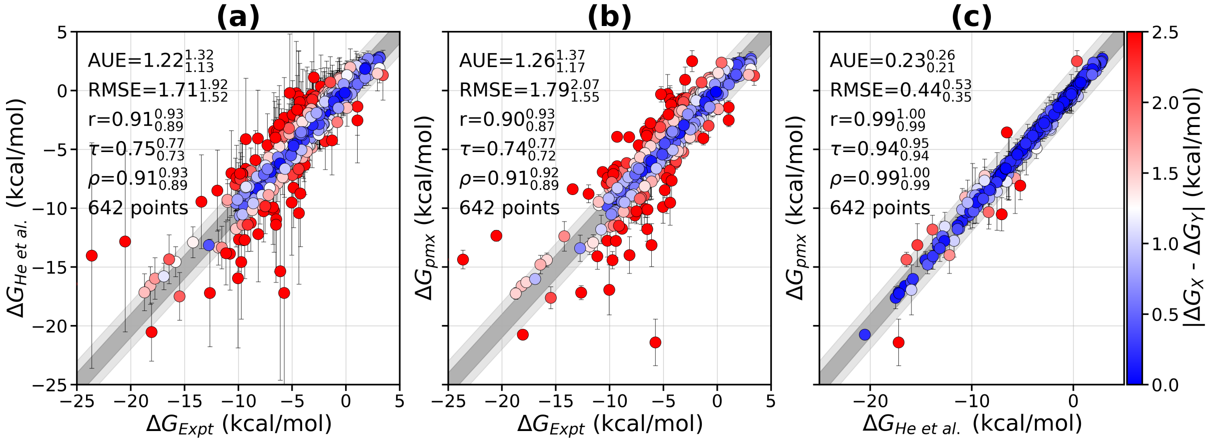

Figure S1: Scatter plots comparing HFEs estimated using the GAFF2/AM1-BCC approach by He *et al.* and by the current study (pmx) are shown against experiments, and against each other, for all the 642 solutes. The darker and lighter grey shades represent values within 1 and 2 kcal/mol of the experiments, respectively. The r, τ, and ρ are the Pearson's r, Kendall's τ, and Spearman's ρ, respectively.

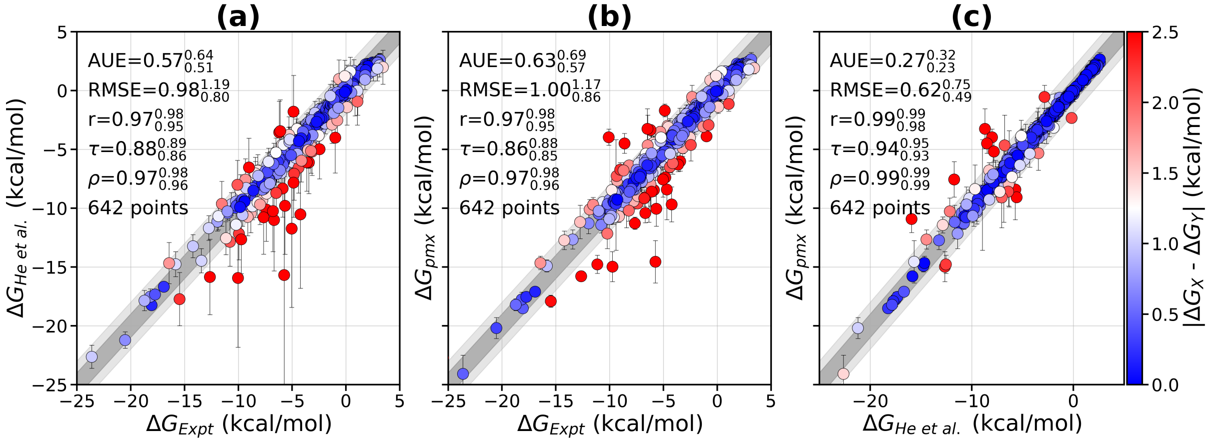

Figure S2: Scatter plots comparing HFEs estimated using the GAFF2/ABCG2 approach by He *et al.* and by the current study (pmx) are shown against experiments, and against each other, for all the 642 solutes.

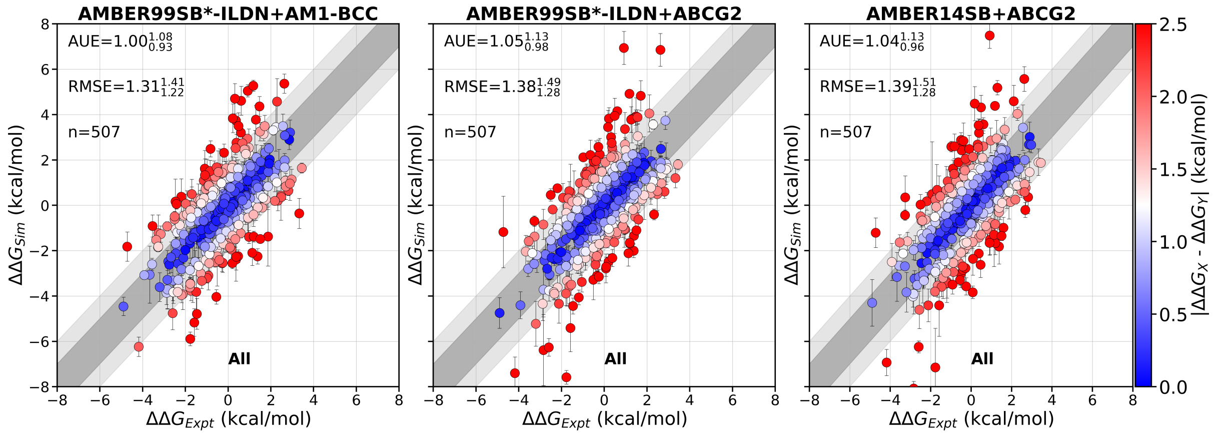

Figure S3: The scatter plots of estimated $\Delta\Delta G$ against experiments, and against each other, for all the ligand transformations from 12 targets. The darker and lighter grey shades represent within 1 and 2 kcal/mol from experiments, respectively.

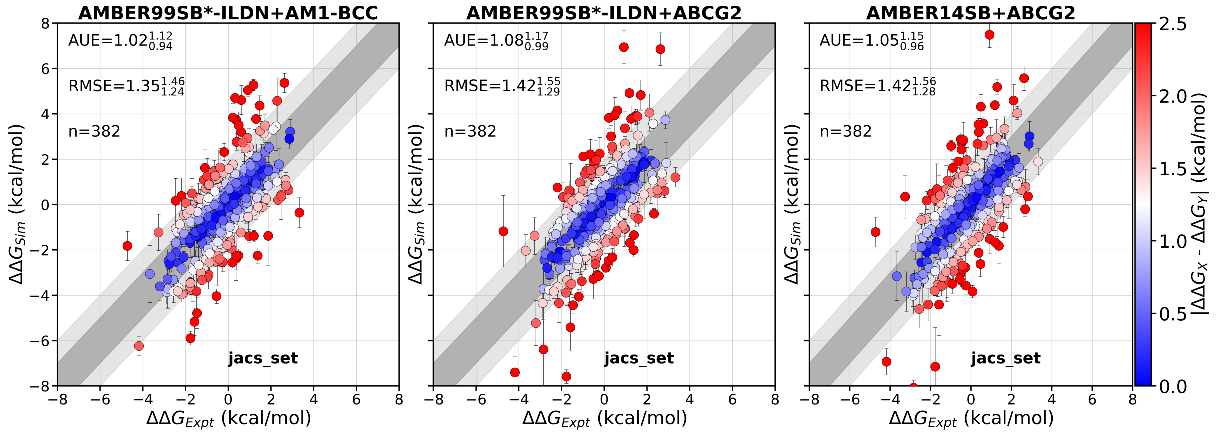

Figure S4: Similar plots as Figure S3, but only for the targets from 'jacs_set'.[7]

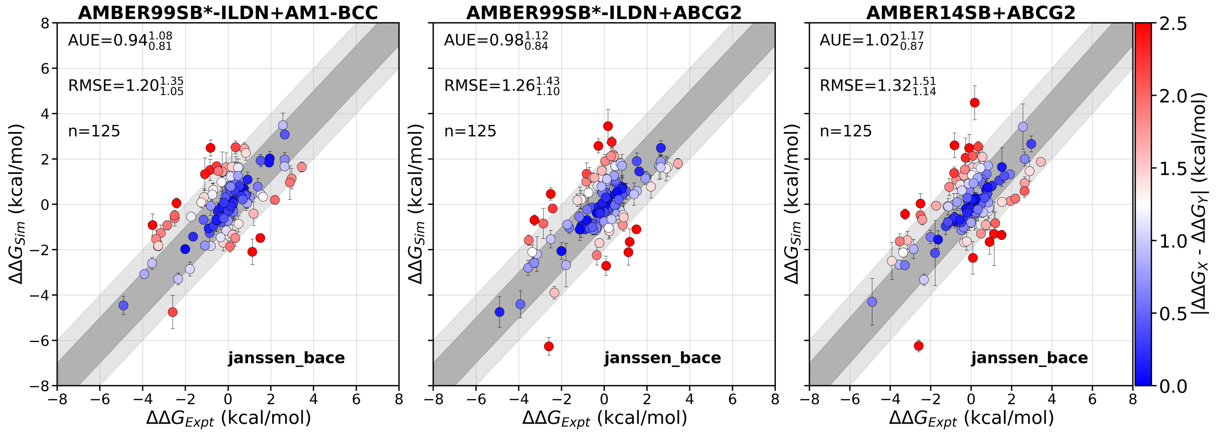

Figure S5: Similar plots as Figure S3, but only for the targets from 'janssen_bace'[8,9] set.

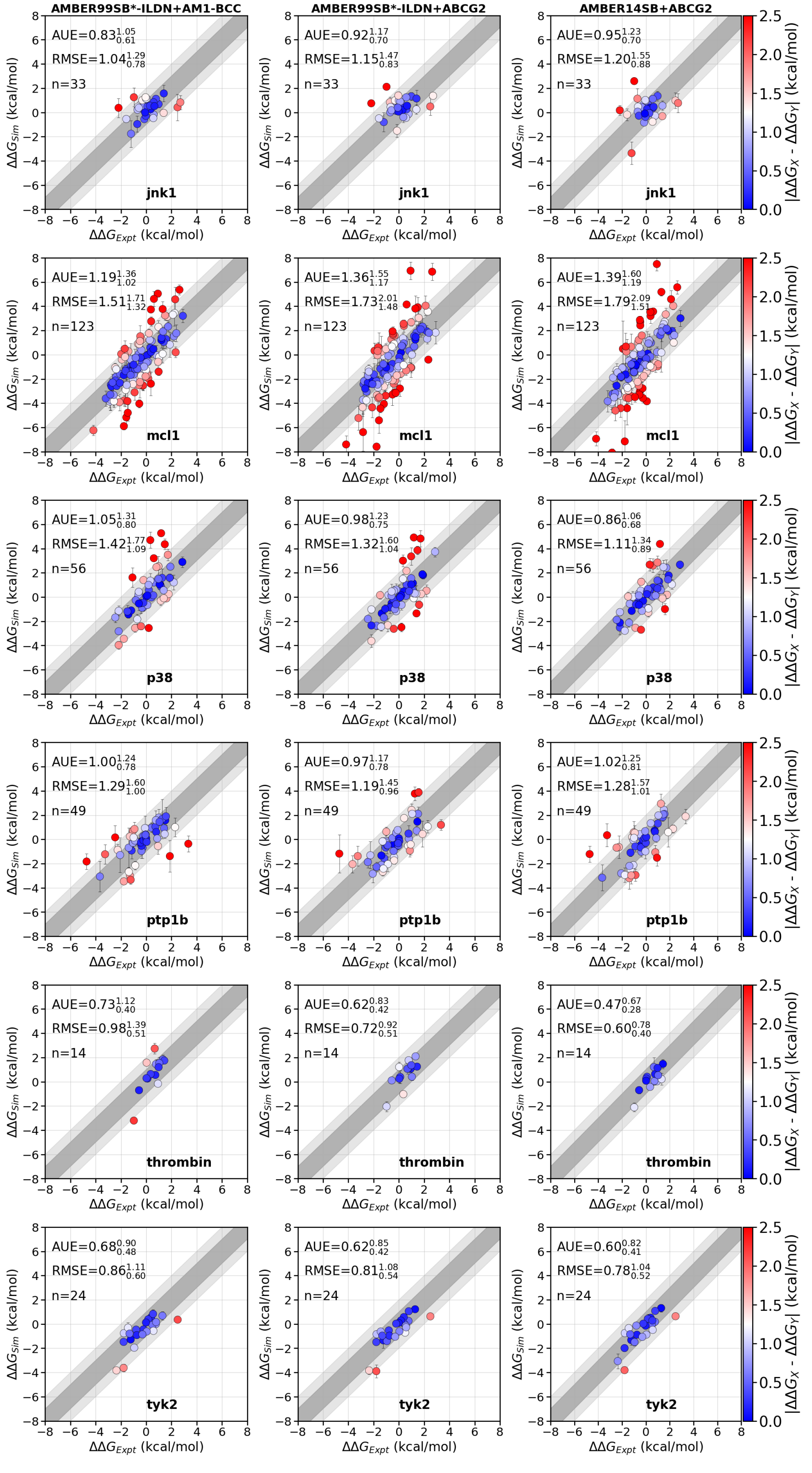

Figure S6: The scatter plots of estimated $\Delta\Delta G$ against experiments, and against each other, for 6 targets plotted individually. The targets' names are written at the bottom.

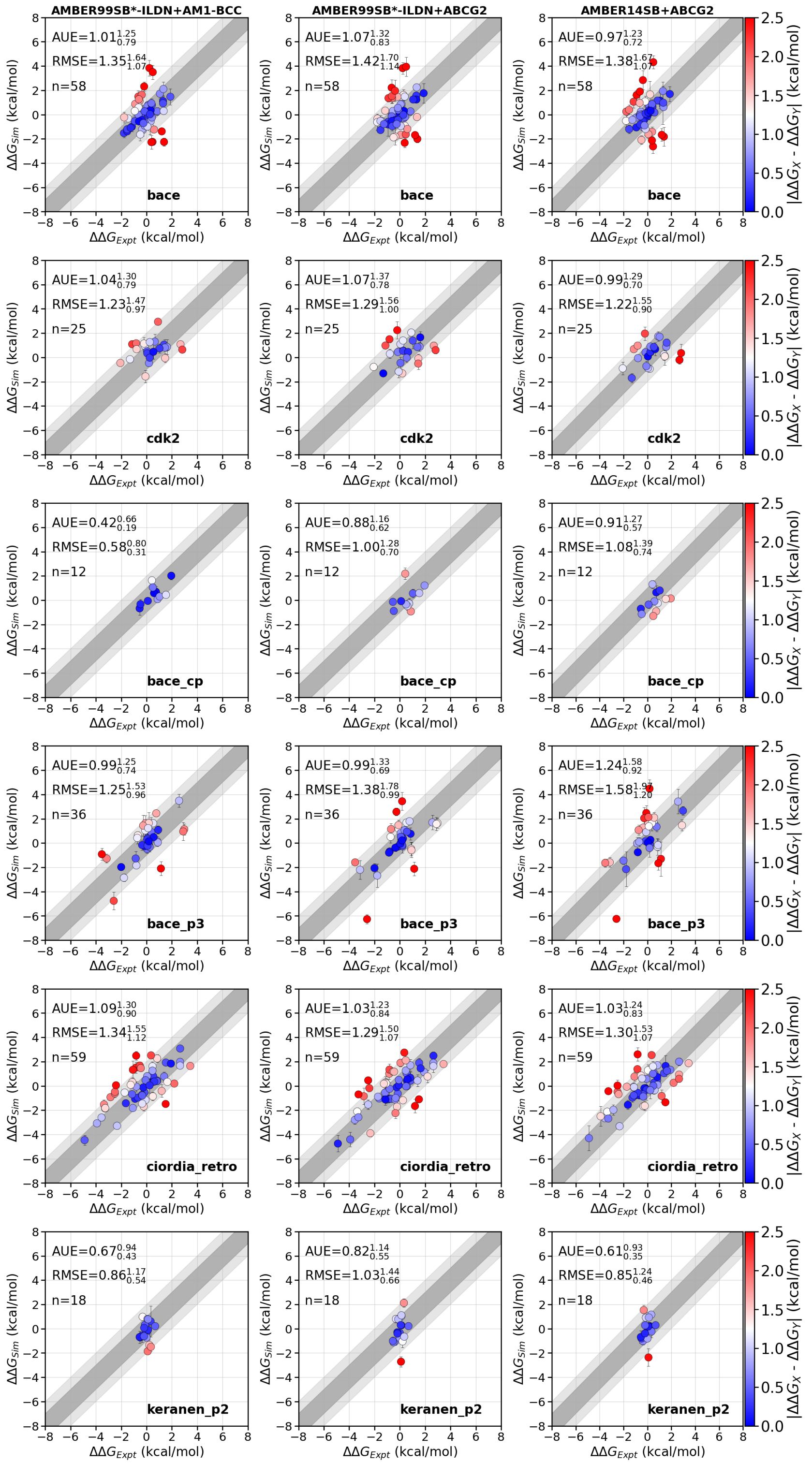

Figure S7: The scatter plots of estimated $\Delta\Delta G$ against experiments, and against each other, for the remaining 6 targets plotted individually. The targets' names are written on the bottom. "bace_cp" and "bace_p3" are abbreviations for "bace_ciordia_prospective" and "bace_p3_arg368_in", respectively.

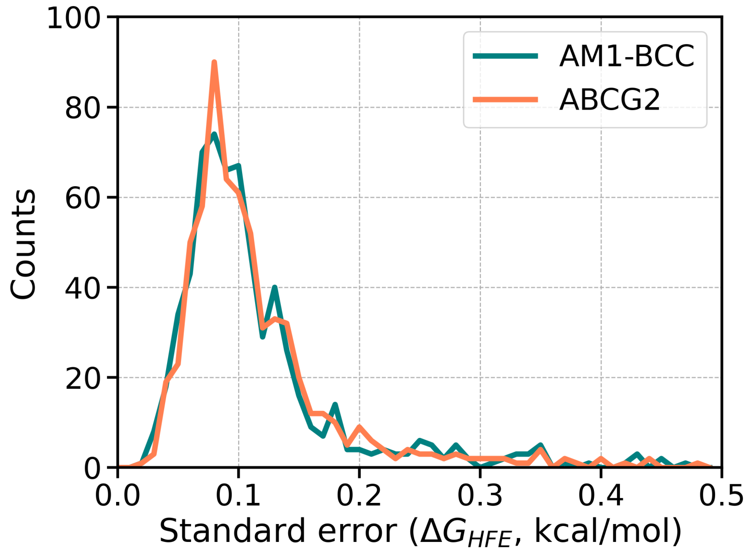

Figure S8: The distribution of the standard error on ΔG_{HFE} (hydration free energy), estimated using the protocol described in section S1.3.

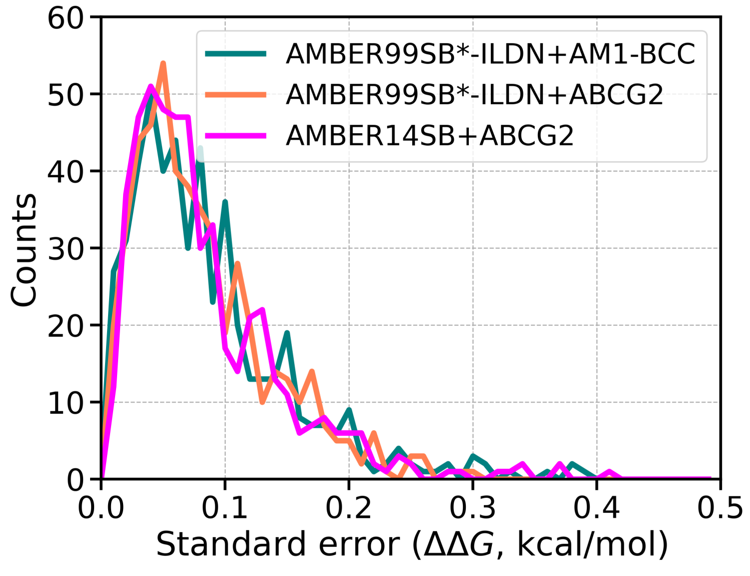

Figure S9: The distribution of the standard error on $\Delta\Delta G$ (protein-ligand RBFE), estimated using the protocol described in section S1.3.

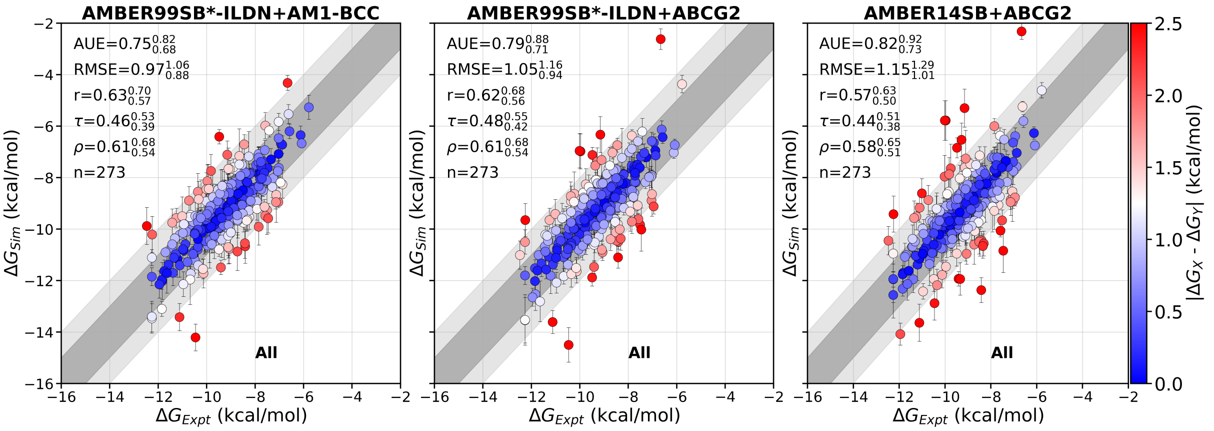

Figure S10: The scatter plots of estimated ΔG against experiments, and against each other, for all the ligands from 12 targets. The darker and lighter grey shades represent values within 1 and 2 kcal/mol of the experiments, respectively. The correlation coefficients are computed as weighted averages of the values obtained for individual targets.

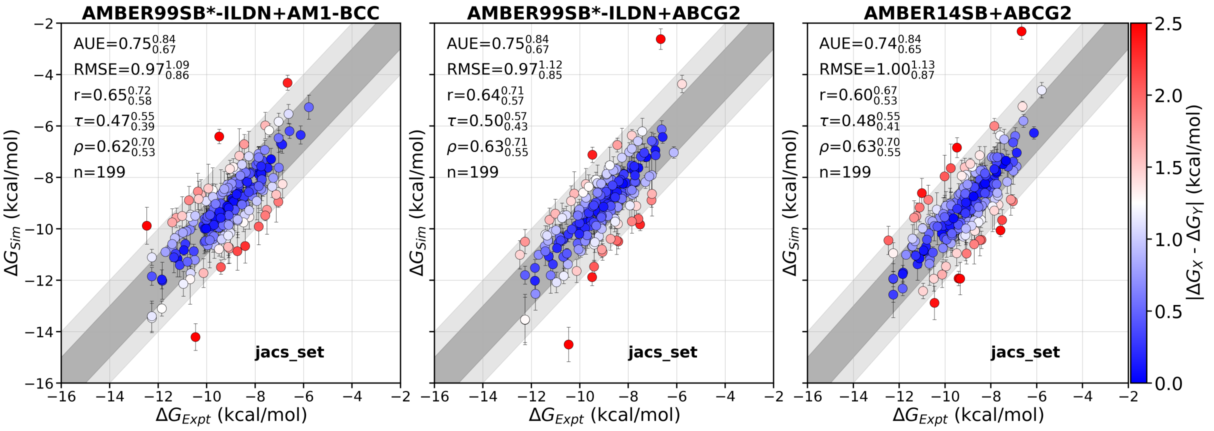

Figure S11: Similar plots as Figure S10, but only for the targets from 'jacs_set'.[7]

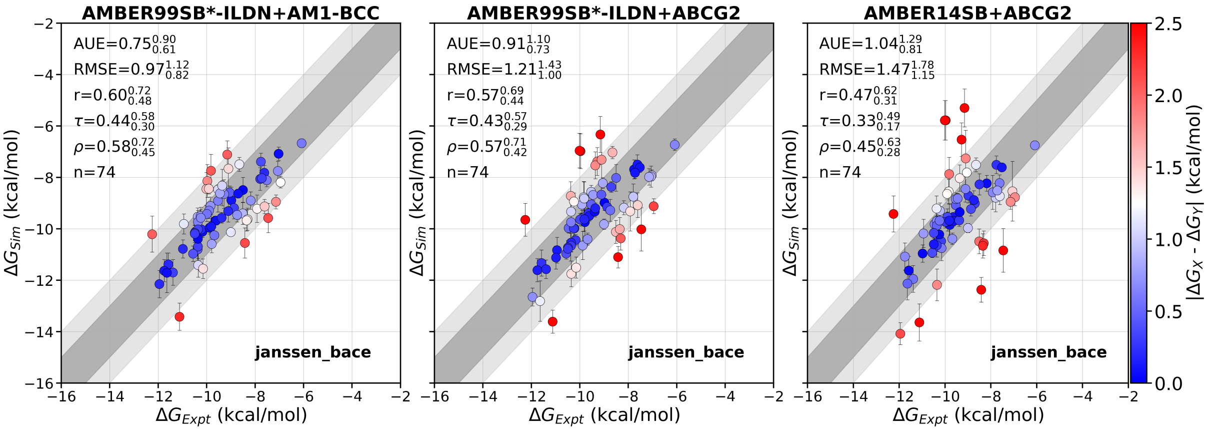

Figure S12: Similar plots as Figure S10, but only for the targets from 'janssen_bace'[8,9] set.

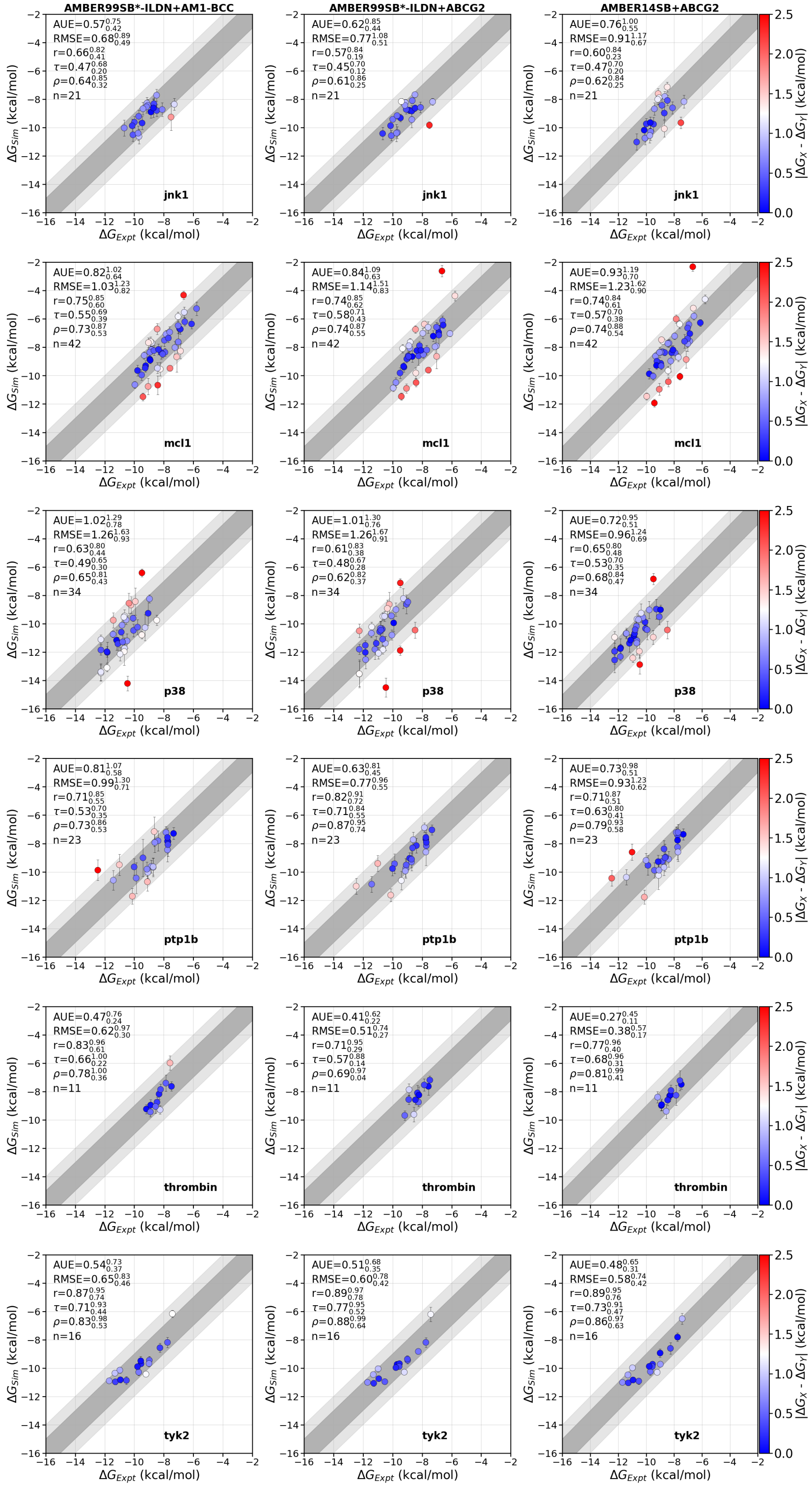

Figure S13: The scatter plots of estimated ΔG against experiments, and against each other, for 6 targets plotted individually. The targets' names are written at the bottom.

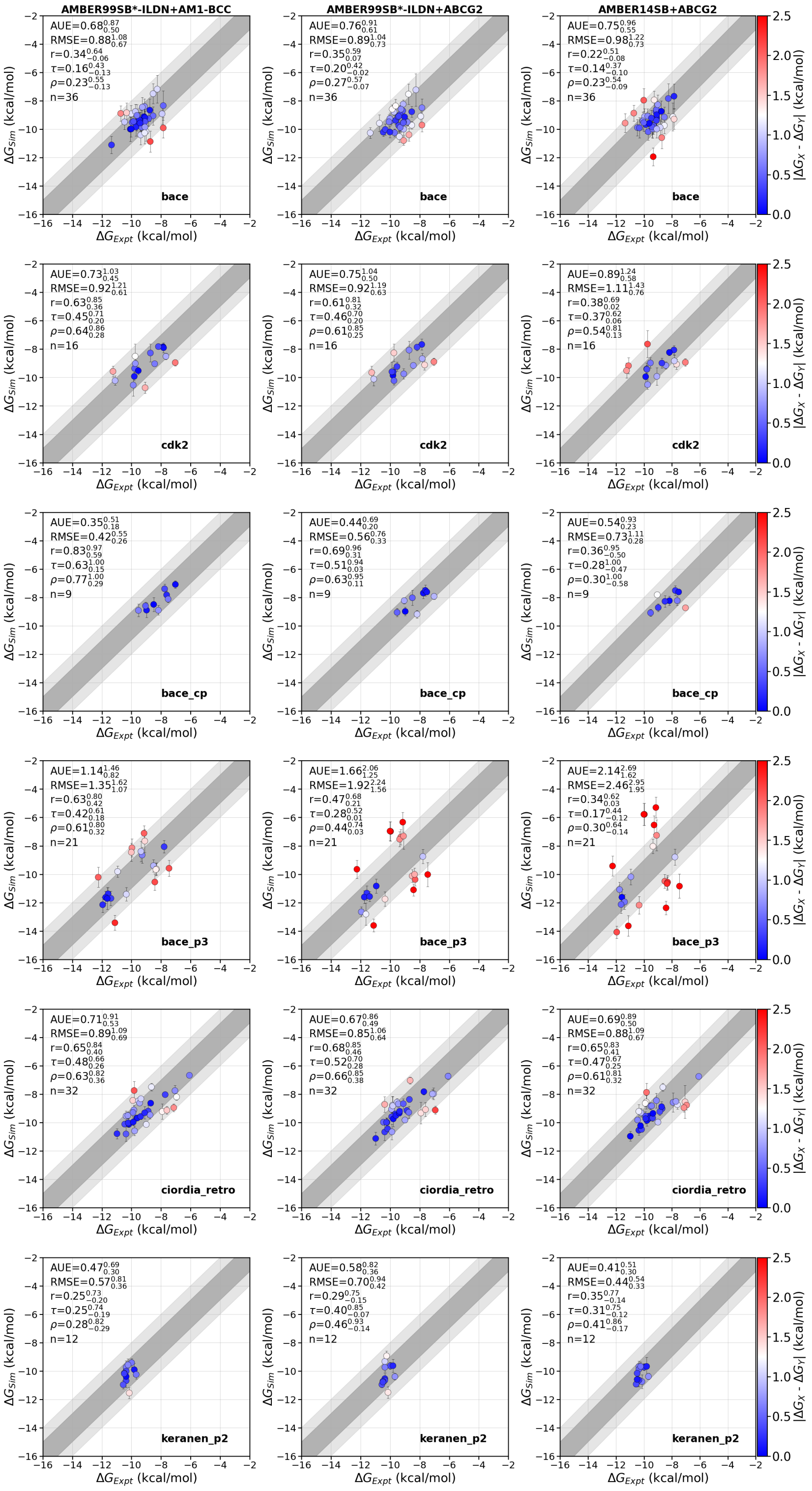

Figure S14: The scatter plots of estimated ΔG against experiments, and against each other, for the remaining 6 targets plotted individually. The targets' names are written on the bottom. "bace_cp" and "bace_p3" are abbreviations for "bace_ciordia_prospective" and "bace_p3_arg368_in", respectively.

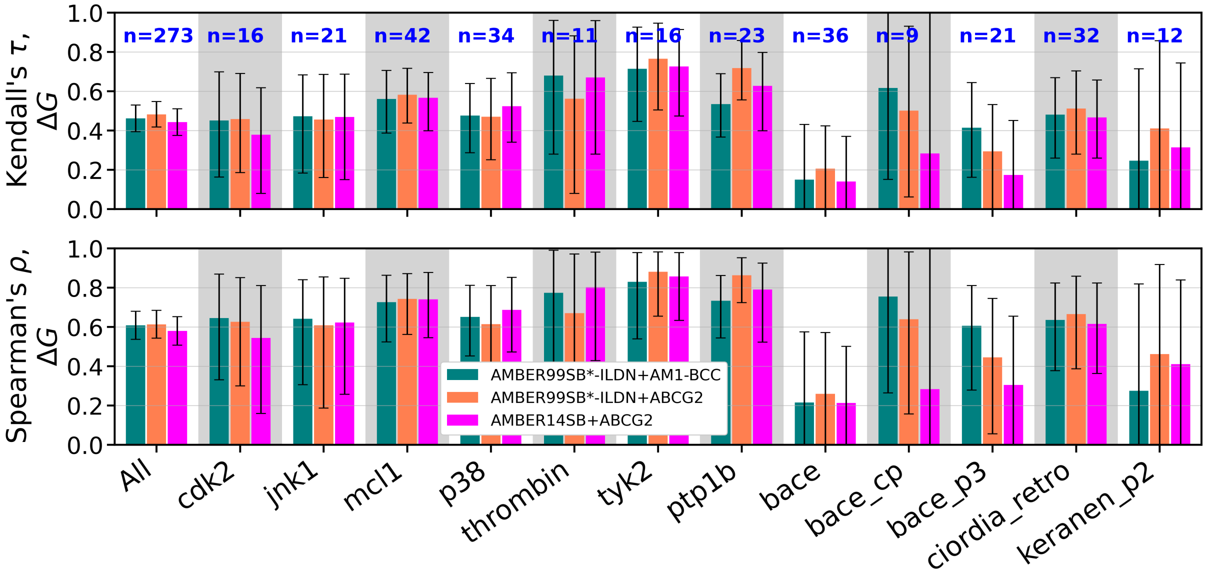

Figure S15: Correlation (Kendall's τ and Spearman's ρ) between the simulated ΔG (estimated from $\Delta\Delta G$) and experiments. The Pearson's r is presented in Figure 2c. "bace_cp" and "bace_p3" are abbreviations for "bace_ciordia_prospective" and "bace_p3_arg368_in", respectively.

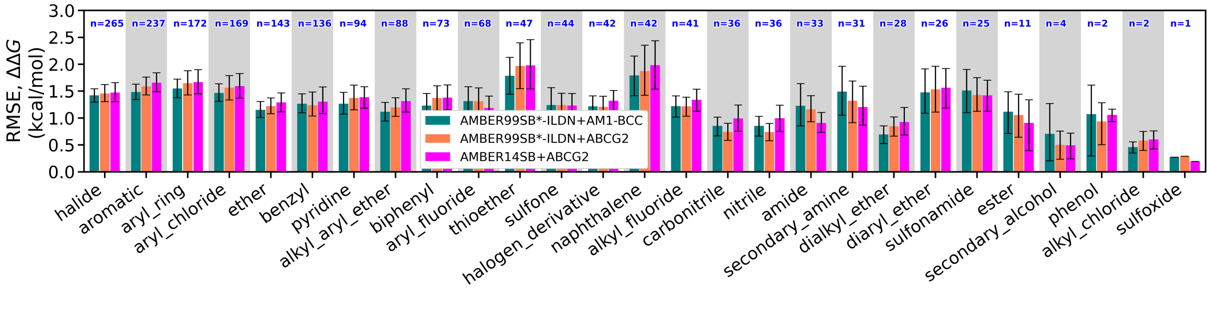

Figure S16: RMSEs for subsets of ligand transformations involving different functional groups, highlighting those groups where the RMSE difference between AMBER99SB*-ILDN+AM1-BCC and AMBER99SB*-ILDN+ABCG2 charge methods is <= 1 kJ/mol (0.24 kcal/mol). The groups with RMSE difference of > 1 kJ/mol are shown in Figure 2a.

Table S1: RMSE between experimental and simulated $\Delta\Delta G$ with 95% confidence interval (subscript and superscript) for all the targets.

**Target**	**n (edges)**	**AMBER99SB*-ILDN +AM1-BCC**	**AMBER99SB*-ILDN +ABCG2**	**AMBER14SB +ABCG2**
All	507	$1.31_{1.22}^{1.41}$	$1.38_{1.28}^{1.49}$	$1.39_{1.28}^{1.51}$
cdk2	25	$1.23_{0.97}^{1.46}$	$1.29_{1.00}^{1.56}$	$1.22_{0.89}^{1.55}$
jnk1	33	$1.04_{0.79}^{1.29}$	$1.15_{0.85}^{1.47}$	$1.21_{0.88}^{1.55}$
mcl1	123	$1.51_{1.31}^{1.71}$	$1.74_{1.49}^{2.01}$	$1.79_{1.52}^{2.09}$
p38	56	$1.42_{1.10}^{1.76}$	$1.32_{1.05}^{1.61}$	$1.11_{0.89}^{1.34}$
thrombin	14	$0.98_{0.51}^{1.39}$	$0.72_{0.51}^{0.92}$	$0.60_{0.39}^{0.78}$
tyk2	24	$0.85_{0.59}^{1.11}$	$0.81_{0.54}^{1.08}$	$0.78_{0.52}^{1.03}$
ptp1b	49	$1.29_{1.01}^{1.59}$	$1.19_{0.95}^{1.45}$	$1.28_{1.01}^{1.58}$
bace	58	$1.35_{1.06}^{1.63}$	$1.42_{1.14}^{1.70}$	$1.37_{1.07}^{1.67}$
bace_cp	12	$0.58_{0.32}^{0.80}$	$0.99_{0.70}^{1.28}$	$1.08_{0.75}^{1.38}$
bace_p3	36	$1.25_{0.97}^{1.53}$	$1.38_{0.99}^{1.76}$	$1.58_{1.20}^{1.98}$
ciordia_retro	59	$1.34_{1.12}^{1.55}$	$1.29_{1.08}^{1.50}$	$1.30_{1.07}^{1.53}$
keranen_p2	18	$0.86_{0.53}^{1.16}$	$1.02_{0.66}^{1.43}$	$0.85_{0.47}^{1.25}$

Table S2: RMSE between experimental and simulated ΔG with 95% confidence interval (subscript and superscript) for all the targets.

**Target**	**n (ligands)**	**AMBER99SB*-ILDN +AM1-BCC**	**AMBER99SB*-ILDN +ABCG2**	**AMBER14SB +ABCG2**
All	273	$0.97_{0.88}^{1.07}$	$1.05_{0.94}^{1.16}$	$1.15_{1.01}^{1.29}$
cdk2	16	$0.93_{0.62}^{1.22}$	$0.92_{0.63}^{1.19}$	$1.12_{0.76}^{1.43}$
jnk1	21	$0.68_{0.50}^{0.89}$	$0.77_{0.51}^{1.08}$	$0.91_{0.66}^{1.16}$
mcl1	42	$1.03_{0.82}^{1.23}$	$1.14_{0.83}^{1.51}$	$1.23_{0.89}^{1.62}$
p38	34	$1.26_{0.92}^{1.63}$	$1.26_{0.92}^{1.66}$	$0.96_{0.68}^{1.24}$
thrombin	11	$0.63_{0.29}^{0.97}$	$0.51_{0.28}^{0.73}$	$0.38_{0.17}^{0.56}$
tyk2	16	$0.65_{0.46}^{0.83}$	$0.61_{0.42}^{0.78}$	$0.58_{0.42}^{0.74}$
ptp1b	23	$0.99_{0.71}^{1.29}$	$0.77_{0.55}^{0.97}$	$0.93_{0.62}^{1.24}$
bace	36	$0.88_{0.67}^{1.09}$	$0.89_{0.73}^{1.04}$	$0.98_{0.73}^{1.22}$
bace_cp	9	$0.42_{0.26}^{0.55}$	$0.56_{0.33}^{0.76}$	$0.73_{0.28}^{1.12}$
bace_p3	21	$1.35_{1.07}^{1.61}$	$1.91_{1.56}^{2.24}$	$2.47_{1.96}^{2.95}$
ciordia_retro	32	$0.89_{0.69}^{1.08}$	$0.85_{0.63}^{1.05}$	$0.89_{0.67}^{1.09}$
keranen_p2	12	$0.57_{0.36}^{0.81}$	$0.70_{0.43}^{0.95}$	$0.44_{0.34}^{0.55}$

Table S3: Pearson's r between experimental and simulated ΔG, with 95% confidence interval (subscript and superscript) for all the targets.

**Target**	**n (ligands)**	**AMBER99SB*-ILDN +AM1-BCC**	**AMBER99SB*-ILDN +ABCG2**	**AMBER14SB +ABCG2**
All	273	$0.63^{0.70}_{0.57}$	$0.62^{0.68}_{0.56}$	$0.57^{0.63}_{0.50}$
cdk2	16	$0.63^{0.85}_{0.36}$	$0.61^{0.82}_{0.31}$	$0.39^{0.69}_{0.06}$
jnk1	21	$0.66^{0.83}_{0.41}$	$0.58^{0.83}_{0.18}$	$0.60^{0.84}_{0.24}$
mcl1	42	$0.75^{0.85}_{0.60}$	$0.74^{0.84}_{0.62}$	$0.74^{0.84}_{0.61}$
p38	34	$0.63^{0.79}_{0.44}$	$0.61^{0.82}_{0.35}$	$0.65^{0.81}_{0.48}$
thrombin	11	$0.84^{0.96}_{0.61}$	$0.72^{0.95}_{0.27}$	$0.77^{0.96}_{0.35}$
tyk2	16	$0.87^{0.95}_{0.74}$	$0.89^{0.96}_{0.78}$	$0.89^{0.95}_{0.76}$
ptp1b	23	$0.71^{0.85}_{0.56}$	$0.83^{0.91}_{0.71}$	$0.71^{0.86}_{0.48}$
bace	36	$0.35^{0.64}_{-0.06}$	$0.34^{0.57}_{0.04}$	$0.23^{0.49}_{-0.06}$
bace_cp	9	$0.84^{0.97}_{0.64}$	$0.69^{0.95}_{0.33}$	$0.35^{0.94}_{-0.52}$
bace_p3	21	$0.63^{0.79}_{0.41}$	$0.47^{0.70}_{0.23}$	$0.34^{0.61}_{0.03}$
ciordia_retro	32	$0.65^{0.83}_{0.38}$	$0.69^{0.86}_{0.47}$	$0.65^{0.84}_{0.40}$
keranen_p2	12	$0.25^{0.73}_{-0.21}$	$0.28^{0.74}_{-0.15}$	$0.35^{0.76}_{-0.16}$

Table S4: p value obtained using a paired Student's t-test on $\Delta\Delta G$ estimates obtained with different force field combinations.

**Target**	**n (edges)**	**AMBER99SB*-ILDN +AM1-BCC vs AMBER99SB*-ILDN+ABCG2**	**AMBER99SB*-ILDN +AM1-BCC vs AMBER14SB +ABCG2**	**AMBER99SB*-ILDN +ABCG2 vs AMBER14SB +ABCG2**
All	507	0.21	0.40	0.69
cdk2	25	0.32	0.05	0.11
jnk1	33	0.37	0.56	0.13
mcl1	123	0.56	0.94	0.43
p38	56	0.10	0.15	0.88
thrombin	14	0.85	0.03	0.04
tyk2	24	0.89	0.28	0.22
ptp1b	49	0.88	0.90	0.99
bace	58	0.34	0.35	0.80
bace_cp	12	0.05	0.02	0.23
bace_p3	36	0.80	0.06	0.02
ciordia_retro	59	0.17	0.17	0.94
keranen_p2	18	0.59	0.19	0.41

Table S5: p value obtained using a paired Student's t-test on $\Delta\Delta G$ estimates for perturbations involving different functional groups highlighted in Figure 2a.

**Target**	**n (edges)**	**AMBER99SB*-ILDN +AM1-BCC vs AMBER99SB*-ILDN+ABCG2**	**AMBER99SB*-ILDN +AM1-BCC vs AMBER14SB +ABCG2**	**AMBER99SB*-ILDN +ABCG2 vs AMBER14SB +ABCG2**
pyrrole	47	0.86	0.79	0.90
thiophene	37	0.92	0.83	0.86
aryl_bromide	27	0.78	0.48	0.22
furan	18	0.12	0.01	0.32
primary_alcohol	16	0.11	0.20	0.79
aniline	15	0.47	0.83	0.51
alkyne	15	0.84	0.26	0.16
tertiary_amine	13	0.54	0.83	0.56
tertiary_amide	11	0.77	0.92	0.87
quinoline	6	0.40	0.07	0.94
aryl_iodide	4	0.63	0.11	0.23
tertiary_alcohol	3	0.91	0.99	0.66

References

(1) Mobley, D. L.; Guthrie, J. P. FreeSolv: a database of experimental and calculated hydration free energies, with input files. *Journal of Computer-Aided Molecular Design* **2014**, *28*, 711–720.

(2) Landrum, G. et al. rdkit/rdkit: 2020_09_1 (Q3 2020) Release. 2020; `https://doi.org/10.5281/zenodo.4107869`.

(3) Wang, J.; Wang, W.; Kollman, P. A.; Case, D. A. Automatic atom type and bond type perception in molecular mechanical calculations. *Journal of Molecular Graphics and Modelling* **2006**, *25*, 247–260.

(4) Sousa da Silva, A. W.; Vranken, W. F. ACPYPE-Antechamber python parser interface. *BMC Research Notes* **2012**, *5*, 1–8.

(5) He, X.; Man, V. H.; Yang, W.; Lee, T.-S.; Wang, J. ABCG2: A Milestone Charge Model for Accurate Solvation Free Energy Calculation. *Journal of Chemical Theory and Computation* **2025**, *21*, 3032–3043.

(6) Joung, I. S.; Cheatham III, T. E. Determination of alkali and halide monovalent ion parameters for use in explicitly solvated biomolecular simulations. *The Journal of Physical Chemistry B* **2008**, *112*, 9020–9041.

(7) Wang, L. et al. Accurate and reliable prediction of relative ligand binding potency in prospective drug discovery by way of a modern free-energy calculation protocol and force field. *Journal of the American Chemical Society* **2015**, *137*, 2695–2703.

(8) Ciordia, M.; Pérez-Benito, L.; Delgado, F.; Trabanco, A. A.; Tresadern, G. Application of free energy perturbation for the design of BACE1 inhibitors. *Journal of Chemical Information and Modeling* **2016**, *56*, 1856–1871.

(9) Keranen, H.; Pérez-Benito, L.; Ciordia, M.; Delgado, F.; Steinbrecher, T. B.; Oehlrich, D.; Van Vlijmen, H. W.; Trabanco, A. A.; Tresadern, G. Acylguanidine beta secretase 1 inhibitors: a combined experimental and free energy perturbation study. *Journal of Chemical Theory and Computation* **2017**, *13*, 1439–1453.

(10) Gowers, R. J.; Alibay, I.; Swenson, D. W.; Henry, M. M.; Ries, B.; Baumann, H. M.; Eastwood, J. R. B. The Open Free Energy library. 2023; `https://doi.org/10.5281/zenodo.8344248`.

(11) `https://github.com/OpenFreeEnergy/IndustryBenchmarks2024/tree/main`.

(12) Ross, G. A.; Lu, C.; Scarabelli, G.; Albanese, S. K.; Houang, E.; Abel, R.; Harder, E. D.; Wang, L. The maximal and current accuracy of rigorous protein-ligand binding free energy calculations. *Communications Chemistry* **2023**, *6*, 222.

(13) Gapsys, V.; Michielssens, S.; Seeliger, D.; de Groot, B. L. pmx: Automated protein structure and topology generation for alchemical perturbations. *Journal of Computational Chemistry* **2015**, *36*, 348–354.

(14) Lindorff-Larsen, K.; Piana, S.; Palmo, K.; Maragakis, P.; Klepeis, J. L.; Dror, R. O.; Shaw, D. E. Improved side-chain torsion potentials for the Amber ff99SB protein force field. *Proteins: Structure, Function, and Bioinformatics* **2010**, *78*, 1950–1958.

(15) Best, R. B.; Hummer, G. Optimized molecular dynamics force fields applied to the helix-coil transition of polypeptides. *The Journal of Physical Chemistry B* **2009**, *113*, 9004–9015.

(16) Hornak, V.; Abel, R.; Okur, A.; Strockbine, B.; Roitberg, A.; Simmerling, C. Comparison of multiple Amber force fields and development of improved protein backbone parameters. *Proteins: Structure, Function, and Bioinformatics* **2006**, *65*, 712–725.

(17) Maier, J. A.; Martinez, C.; Kasavajhala, K.; Wickstrom, L.; Hauser, K. E.; Simmerling, C. ff14SB: improving the accuracy of protein side chain and backbone parameters from ff99SB. *Journal of Chemical Theory and Computation* **2015**, *11*, 3696–3713.

(18) Gapsys, V.; Pérez-Benito, L.; Aldeghi, M.; Seeliger, D.; Van Vlijmen, H.; Tresadern, G.; De Groot, B. L. Large scale relative protein ligand binding affinities using non-equilibrium alchemy. *Chemical Science* **2020**, *11*, 1140–1152.

(19) Behera, S.; Hahn, D. F.; Wilson, C. J.; Marsili, S.; Tresadern, G.; Gapsys, V.; de Groot, B. L. Quantification of the Impact of Structure Quality on Predicted Binding Free Energy Accuracy. *Journal of Chemical Information and Modeling* **2025**, *65*, 6927–6938.

(20) Shirts, M. R.; Bair, E.; Hooker, G.; Pande, V. S. Equilibrium Free Energies from Nonequilibrium Measurements Using Maximum-Likelihood Methods. *Physical Review Letters* **2003**, *91*, 140601.

(21) Crooks, G. E. Entropy production fluctuation theorem and the nonequilibrium work relation for free energy differences. *Physical Review E* **1999**, *60*, 2721.

(22) Fletcher, R.; Reeves, C. M. Function minimization by conjugate gradients. *The Computer Journal* **1964**, *7*, 149–154.

(23) Parrinello, M.; Rahman, A. Polymorphic transitions in single crystals: A new molecular dynamics method. *Journal of Applied Physics* **1981**, *52*, 7182–7190.

(24) Van Gunsteren, W. F.; Berendsen, H. J. A leap-frog algorithm for stochastic dynamics. *Molecular Simulation* **1988**, *1*, 173–185.

(25) Hess, B.; Bekker, H.; Berendsen, H. J.; Fraaije, J. G. LINCS: a linear constraint solver for molecular simulations. *Journal of Computational Chemistry* **1997**, *18*, 1463–1472.

(26) Darden, T.; York, D.; Pedersen, L. Particle mesh Ewald: An N log (N) method for Ewald sums in large systems. *The Journal of Chemical Physics* **1993**, *98*, 10089–10092.

(27) Gapsys, V.; Seeliger, D.; de Groot, B. L. New soft-core potential function for molecular dynamics based alchemical free energy calculations. *Journal of Chemical Theory and Computation* **2012**, *8*, 2373–2382.

(28) Abraham, M. J.; Murtola, T.; Schulz, R.; Páll, S.; Smith, J. C.; Hess, B.; Lindahl, E. GROMACS: High performance molecular simulations through multi-level parallelism from laptops to supercomputers. *SoftwareX* **2015**, *1*, 19–25.
